# Advanced transport systems: the future is sustainable and technology-enabled

**DOI:** 10.1038/s41598-024-59438-0

**Published:** 2024-04-24

**Authors:** Yue Cao, Sybil Derrible, Michela Le Pira, Haiping Du

**Affiliations:** 1 School of Cyber Science and Engineering, Wuhan University, Wuhan, 430072 Hubei China; 2 Department of Civil, Materials, and Environmental Engineering, University of Illinois Chicago, 1200 West Harrison St., Chicago, IL 60607 USA; 3 Department of Civil Engineering and Architecture, University of Catania, Via Santa Sofia 64, 95123 Catania, Italy; 4 School of Electrical, Computer and Telecommunications Engineering, University of Wollongong, Northfields Ave, Wollongong, NSW 2522 Australia

**Keywords:** Civil engineering, Electrical and electronic engineering

## Abstract

Transport has always played a major role in shaping society. By enabling or restricting the movement of people and goods, the presence or absence of transport services and infrastructure has historically been determining for cultures to connect, for knowledge to be shared, and for societies to evolve and prosper, or, in contrast, for societies to decay and fail. Since the beginning of the twenty-first century, transport has been going through a revolution worldwide. One of the primary goals for the transport sector is clear: it needs to be decarbonized and become more sustainable. At the same time, technological advances are shaping the transport sector toward smart services and societies. The Special Collection showcases some of the latest advances in research towards sustainable and technology-enabled transport.

## Introduction

The transport sector is fundamental to promoting human development and economic growth. Yet, it is also one of the most impacting and energy-consuming sectors, accounting for a quarter of global energy-related CO_2_ emissions^1^. This is largely because oil products still made up more than 90% of the energy used in transport by 2022^2,3^. The transport sector is also responsible for many other externalities, from social exclusion to crashes, and it is one of the most cost-intensive sectors in terms of public administration^4^.

Right now, the urgency to decarbonize and make transport more sustainable is clear. This is apparent from the articles published in the Special Collection. On purpose, we (the editors) had kept the call for the Special Collection broad by naming it “Advanced Transportation Systems”, but many submissions directly address the need for the transport sector to reduce its carbon footprint, whether by tackling traffic congestion, by making way for electric vehicles, or by promoting alternative travel modes. The first theme that emerged from the Special Collection is therefore sustainability.

The second theme that emerged from the collection is technology. Most submissions either study a technology or use advanced data science techniques to answer their research questions. This emphasis on technology was expected. Artificial Intelligence (AI) and ubiquitous sensing and computing have pervaded virtually every domain, including transport, towards Intelligent Transport Systems (ITS). From technology-enabled crowdsourced transit service to autonomous vehicles and freight delivery, the collection sees much promise in technology.

This editorial synthesizes the key topics and findings of the Special Collection “Advanced Transportation Systems” along the two themes found, and it lays the path for future research in transport.

## Advances toward technology-enabled transport

The application of AI in transport has been growing significantly. As of this writing, typical use cases include autonomous vehicles, drones delivering packages, and sophisticated systems managing complex logistics delivery networks^1^. One report^5^ projected that global AI in the transport market reached $3.5 billion by 2023, an impressive growth rate of 16.5%.

For example, as a fundamental component of autonomous driving systems, environmental perception^6^ enables vehicles to comprehend their surroundings and make intelligent decisions based on this perception. Autonomous Vehicles (AVs) make wise decisions about speed, direction, and safety by recognizing pedestrians, other vehicles, and traffic signs. This capability is crucial for ensuring safe and efficient road navigation^7^. As another killer application, the usage of drones rapidly increased during the COVID-19 pandemic. In the United States, the Alphabet-owned drone delivery company Wing saw demand for its services double, thanks to the drones bringing contactless ways to access consumer goods^8^.

Digital-twin, federated learning, reinforcement learning, and machine learning have been widely applied in the literature and in this Special Collection, ranging from passenger demand forecasting and the prediction of electricity consumption using traffic volume data^9^ to the optimization of traffic signal controls and the evaluation of the pedestrian level of service^10–12^. The debate around the potential of big data analytics is lively, and how/if they will replace traditional transport modelling techniques^13^.

ITS is a holistic system employed in transport management, including information, communication, sensing, electronic control, AI, and computer technologies. ITS provides comprehensive, real-time, accurate, efficient transport and management capabilities to service citizens and operate the city efficiently, such as traffic control, disaster management, and driver monitoring. With the help of ICT and the continuous development of ITS, smart parking has also been upgraded. Compared with traditional parking, smart parking alleviates users from finding available parking spots by notifying users of available spots in advance. Emerging ICT has been integrated with smart parking services, such as using RFID or magnetic sensors to monitor the utilization of parking space, or developing middleware for urban level parking management^14^.

## Advances toward sustainable transport

Decarbonization of the transport sector is an important pathway to climate-change mitigation and presents the potential for future lower emissions. Electric vehicles (EVs) are regarded as a promising solution to achieve intelligent and green transport. With energy cost decreasing and user experience improving continuously, EVs are gaining significant market share. Considering the numerous advantages of EVs, many governments and large organizations are actively engaged in the process of promoting EV industry development^15^. Driven by these factors, over 6.8 million EVs were sold worldwide in 2021, despite supply chain bottlenecks and the then ongoing COVID-19 pandemic. Based on the analysis from Net Zero Emissions by 2050 Scenario, the number of EVs will reach over 300 million in 2030 and 60% of new car sales will be EVs.

Along with this, there has been substantial research on decarbonization of transport system, such as the work in^16–18^ on reduction of vehicle emission, investigating the relationship between electricity consumed at building with travel demand and assessing the impact of on-demand public transit systems considering EVs. Of course, due to the existing drive-by-wire design and in-vehicle system, EVs have more advantages on autonomous technology implementation. Therefore, the application of autonomous EVs is progressively supplanting traditional ICE-based AVs.

Among transport externalities, safety represents one of the big concerns of modern societies. According to the statistic from World Health Organization (WHO)^19^, road traffic crashes result in the deaths of approximately 1.19 million people around the world each year and leave between 20 and 50 million people with non-fatal injuries. More than half of all road traffic deaths occur among vulnerable road users, such as pedestrians, cyclists and motorcyclists. This stems from multiple factors, including scarce road maintenance, pointing to the need to plan an *ad-hoc* planning and scheduling of interventions minimizing road congestion and discomfort^20^. Here, enabling an advanced transportation system is able to alleviate the number and severity of traffic crashes through emerging technologies such as traffic control and traffic operations, crash data collection and analyses, safety information and communication systems and safety policy and planning^12^. Yet, identifying and defining appropriate techniques to study safety remains challenging^21^.

## The future of transport research

In the near future, we can see that the two themes present in the Special Collection (i.e.., sustainability and technology) will remain predominant. The threats of climate change are ever present, and they are not expected to lessen. Research efforts will likely continue to study how the transport sector can be decarbonized, notably leveraging technology. EVs and alternative low-carbon transport modes offer some of the best solutions to reduce the carbon footprint of the transport sector^22^. We therefore expect many more research studies to come out that will study the impact of electrifying vehicles both on the transport and the electricity sectors. Besides, due the increasing concerning on cyber-attacks on road infrastructures and automobile, resilience in transport remains a critical topic as well, both on the physical asset such as road resilience as well as cyber-resilience which will likely get more attention as connected and autonomous vehicles become more popular.

Finally, issues related to inequity and social and environmental justice in transport will likely get more attention as they have in other domains. Sustainability issues can be tackled by leveraging on new flexible transport services, which are undoubtedly enabled by technology. The idea to have integrated and multimodal transport systems, accessible by users on-demand and according to their heterogeneous preferences is something that has driven research—more at a theoretical level than a practical one—toward the concept of Mobility as a Service (MaaS). Despite many uncertainties, considering the role that transport plays in society, what is certain is that much more research is needed, making transport research a rich, multidisciplinary and constantly evolving field.
